# Metabolic Signature of Sun Exposed Skin Suggests Catabolic Pathway Overweighs Anabolic Pathway

**DOI:** 10.1371/journal.pone.0090367

**Published:** 2014-03-06

**Authors:** Manpreet Randhawa, Vineet Sangar, Samantha Tucker-Samaras, Michael Southall

**Affiliations:** 1 Johnson & Johnson Skin Research Center, CPPW, a Division of Johnson & Johnson Consumer Companies, Inc., Skillman, New Jersey, United States of America; 2 Institute for Systems Biology, Seattle, Washington, United States of America; University of Tennessee, United States of America

## Abstract

Skin chronically exposed to sun results in phenotypic changes referred as photoaging. This aspect of aging has been studied extensively through genomic and proteomic tools. Metabolites, the end product are generated as a result of biochemical reactions are often studied as a culmination of complex interplay of gene and protein expression. In this study, we focused exclusively on the metabolome to study effects from sun-exposed and sun-protected skin sites from 25 human subjects. We generated a highly accurate metabolomic signature for the skin that is exposed to sun. Biochemical pathway analysis from this data set showed that sun-exposed skin resides under high oxidative stress and the chains of reactions to produce these metabolites are inclined toward catabolism rather than anabolism. These catabolic activities persuade the skin cells to generate metabolites through the salvage pathway instead of *de novo* synthesis pathways. Metabolomic profile suggests catabolic pathways and reactive oxygen species operate in a feed forward fashion to alter the biology of sun exposed skin.

## Introduction

Skin is the largest and one of the most complex organs in human body, accounting for almost 15% of total body weight. It serves as an important environmental interface and thus acts as a first line of defense against various environmental insults. It is a highly metabolically active organ that tries to maintain internal homeostasis by communicating the external signals to internal biological processes [1]. Skin when chronically exposed overtime to sun, not only results in medical conditions like melanoma but also influences its phenotypic appearance typically referred as photo aging [2], [3]. Various physical and chemical environmental stressors that originate from sun exposure or pollution can induce alterations in skin at genetic, protein as well as at the metabolite level. The phenotypic changes often manifest in the skin as pigmentation abnormalities, epidermal thickening, erythema and others. These changes are due to alterations in various biological responses including DNA damage[4] inflammation, immune suppression [5], oxidative stress, hyperplastic responses in skin [6] as well as perturbed hormonal balances[7].

Photoaging is considered of great cosmetic concern and has been studied very thoroughly in terms of phenotypic alterations and corresponding changes in cellular mechanisms in skin. The consequences of sun exposure have been previously documented both at the genetic and proteomic level; however studies of the contribution of sun exposure to biochemical changes that result in alterations in skin metabolome have essentially been limited to a few biomolecules such as glutathione and catecholamine [8]. Glutathione either in oxidized or reduced form, is often discussed in terms of its antioxidant capacity whereas catecholamines metabolism is discussed in detail in the context of pigmentation pathways [9]. Studies on these single classes of metabolites are often discussed in relevance to their biological pathway and are described as an outcome of a particular genetic pathway, yet a holistic approach to understand the effect of a biochemically related group of metabolites is missing. For example biomolecules such as catecholamines and acetylcholine, a class of neurotransmitters produced as a result of external stimuli; can serve as a precursor to biogenic amines [1], [10]. These neurotransmitters can act in a paracrine or autocrine fashion, thus having systemic affect whereas biogenic amines can also initiate plethora of effects[11]. Biomolecules like this emphasize the importance of skin as a neuroendocrine organ and a center for multidirectional communications between different parts of the body [11], [12]. Therefore there is a need to study changes in the metabolomic profile of a multifunctional organ such as skin in a holistic way, as these changes accounts for both negative effects like oxidative stress or hyper proliferative phases [6] as well as modulates the positive effects like acceleration of repair mechanisms in skin.

Metabolites, as we know are considered as the end product of a complex interplay between the changes and interactions at genomic and protein levels. However studies of few metabolites, often examined from sun-exposed skin cells do not provide a full picture of the metabolomic signature in sun-exposed skin. Wide range of metabolic alterations that occur during photoaging can be studied by performing metabolomics; which is a relatively young branch of “omics”. This branch of science focuses on studying (qualitatively, quantitatively or both) the metabolome (the sum of all metabolites in a matrix) that cells or organisms generate under a given particular biological condition. These metabolites could be generated or broken down by the cells, residing in the cells, secreted by the cells or taken up from ECM (Extracelluar Matrix). A metabolomics investigation provides us the ability to assess changes in the abundance of large numbers of metabolites representing multiple classes of compounds and these changes capture global shifts such as catabolic or anabolic metabolism and can present an overall physiological status such as stress or hyperactivity of the biological system. A metabolomic profile is the downstream product of numerous genome-wide or proteome-wide interactions, so it can be a very proximal snapshot of an organism's phenotype. Studying these changes in the context of biochemical networks and pathways has shown great promise as a means to identify biomarkers of disease [13].

Here, we present results of an *in vivo* study focusing on the effects of sunlight on the metabolomic profile of the skin. In this study, we compared biochemical profiles of the biopsies extracted from sun-exposed skin (lower outer arm) to biopsies extracted from sun-protected skin (upper inner arm). The metabolomic comparison from this study indicated that UV exposure *i*) induces catabolism of the biomolecules *ii*) exhibits increased oxidative stress; *iii*) catabolism and oxidative stress operate in a feed forward fashion. This study provided a better understanding of the biological responses of sunexposure at the metabolite level. In addition to recapitulating some of the previously published observations, we have identified other metabolic changes in skin associated with sun exposure that were previously unknown.

## Materials and Methods

### Study design

Twenty-five healthy women aged 40–50 years of Fitzpatrick skin types I and II were recruited for this study in a single center study. We obtained human skin biopsies from the sun-exposed area (volar arm) and the non-exposed area (upper inner arm). Allendale Institutional Review Board approved the study protocol and all study participants signed an informed consent form prior to enrollment. Subjects having moderate to severe photo damaged skin was included in the study and a dermatologist determined the damages. Two four mm full-thickness skin punch biopsy were taken each from the sun-exposed volar arm and from the non-exposed upper inner arm. After immediately removing the subcutaneous fat, the biopsy was carefully frozen in liquid nitrogen and stored at −80°C and later on the samples were analyzed for global untargeted metabolic profiling by Metabolon Inc. (Durham, NC).

### Sample preparation

Automatic MicroLab STAR system from Hamilton Company was used to prepare the samples from metabolomics analysis. Prior to the first step in the extraction process, recovery standards were added for QC purposes. The samples were homogenized by soaking in 80% MeOH to extract the small molecules at a volume/mass ratio of 4∶1 [14]. After homogenization of the samples, a series of organic and aqueous extractions were performed to remove the protein fraction while allowing maximum recovery of small molecules. The resulting extract was then divided into two fractions; one for analysis by Liquid chromatography (LC) and one for analysis by Gas chromatography (GC) and the organic sample was removed by putting the sample briefly in TurboVap (Zymark) and subsequently samples were frozen and dried under vacuum. Samples were then prepared for the appropriate instrument, either LC/MS or GC/MS. A “Client Matrix” (CMTRX) sample was generated by combining aliquots of different samples to assess process variability throughout the data set, on all analytical platforms. Periodic injections of these CMTRX samples served as technical replicates. This allowed monitoring and assessment of variability in the quantitation of all consistently detected metabolites and overall process variability and platform performance.

### Sample analysis

Extracts of all experimental and CMTRX samples were split for analysis on the GC/MS and LC/MS/MS platforms. We used the median relative standard deviation (RSD) for the biochemicals consistently measured in the CMTRX to represent the total variability within the process for the actual experimental samples and the variability in quantitation of the endogenous metabolites within these samples. The RSD values for various metabolites ranged from 2.5 to 24.9.

The samples for LC/MS were analyzed on a Waters ACQUITY UPLC (Waters, Millford, MA, USA) using the method described by Evans *et al* [14]. Briefly, the extracts were reconstituted in formic acid and were eluted at the flow rate of 350 µl min^−1^ using (1) a gradient with 0.1% formic acid in water and (2) 0.1% formic acid in methanol (0 to 70% B in 4 min, 70 to 98% B in 0.5 min, 98% B for 0.9 min), while the extracts reconstituted in ammonium bicarbonate were gradient eluted at 350 µl min^−1^ using (1) 6.5 mM ammonium bicarbonate in water, pH 8, and (2) 6.5 mM ammonium bicarbonate in 95∶5 methanol: water (same gradient profile as above). A 5 µl aliquot of sample was injected using 2 times overfill and analyzed using an LTQ mass spectrometer (Thermo Fisher Corp., Waltham, MA, USA) with ESI. The instrument scanned 99–1000 *m*/*z* and alternated between MS and MS/MS scans using dynamic exclusion with an exclusion time of 3 s. The acidic extracts were monitored for positive ions, and the basic extracts were monitored for negative ions in independent injections using separate acid/base dedicated 2.1×100 mm Waters BEH C18 1.7 µm particle columns heated to 40°C.

The samples for GC/MS analysis were re-dried under vacuum desiccation for a minimum of 24 hours and then derivatized using bistrimethyl-silyl-triflouroacetamide (BSTFA) under dried nitrogen. The derivatized samples for GC/MS were analyzed on a Thermo-Finnigan Trace DSQ fast-scanning single-quadrupole MS (Thermo Finnegan, San Jose, CA, USA) operated at unit mass resolving power. The GC column was 20 m×0.18 mm with 0.18 µm film phase consisting of 5% phenyldimethyl silicone, initial oven temperature was 60°C ramped to 340°C in a 16 min period, and helium was the carrier gas. GC/MS was operated using electron impact ionization with a 50 to 750 amu scan range and was tuned and calibrated daily for mass resolution and mass accuracy.

### Data processing

For each chromatogram, raw instrument data was processed by converting retention times (RT) to retention indexes and aligning all samples based on RT markers for the entire LC run. After detecting the ion features, MS signal was integrated using signal to noise threshold, raw MS area threshold, and peak shape criteria. For assessing the quality as well as the differences in metabolites, the individual ion features were grouped based on peak apex retention time and similarly retained ion features. To ensure data quality, Metabolon's established quality control procedures were followed which included instrument performance, chromatography, mass calibration, and extraction efficiency. Coefficients of variation for all standards and processed were checked for each run day. Internal standard retention times and alignment were also checked and validated as a step in quality control. Compounds were identified by automated comparison to reference chemical library entries using software developed for creating library entries from known chemical entities and then automatically fitting those spectra to experimentally derived spectra. Peaks that elute from either the LC or GC method were compared to the library at a particular retention time and its associated spectra for that compound.

Internal standards were primarily used in both the LC and GC methods to calibrate retention times of compounds across all of the samples in the study and for quality control of each instrument run. Identification of known chemical entities was based on comparison with metabolon's library entries of purified external standards.

*Normalization*: Raw area counts for each compound in each sample were normalized to correct for variation resulting from instrument inter-day tuning differences and intraday mass spec performance. Raw area counts for a compound were divided by the median value, setting the medians equal for each day's run. Missing values were assumed to result from areas being below the limits of detection. Missing values for a given compound were imputed with the observed minimum after the normalization step. Quantitative values were derived from integrated raw detector counts of the mass spectrometers. Importantly, while peak area comparisons between samples represent relative amounts of each ion detected, different compounds and ions have different ionization potentials. To preserve all of the variation, yet allow compounds of widely different raw peak areas to be compared directly on a similar graphical scale, the normalized intensities were scaled by their median values for each compound.

### Statistics

Using the normalized data, Matched-pair and Welch's two sample t-tests were performed followed by multiple hypothesis testing to identify significantly different (p<0.05, FDR<0.01) metabolites between the metabolomes from sun-exposed and sun-protected skin samples. Principal component analysis was performed using ‘metabolomics’ package (v 0.1.3) (Livera & Bowne, 2013) in R, a statistical computing environment (www.r-project.org) [R development core team].

*Random Forest Analysis*: Exposed and unexposed were classified using random forest analyses. These analyses provide an estimate of how accurately we can classify the new skin samples in a new data set into exposed and unexposed group. Briefly, this algorithm combines classifiers with low accuracy to generate a classifier with high accuracy. Algorithmically, Random Forests create a set of classification trees based on continual sampling of the experimental units and compounds. Subsequently each observation is then classified based on the majority votes from all of the classification trees. This analysis produces a list of prioritized metabolites with very high classification accuracy [15].

## Results and Discussion

### Global metabolomics profile

**Figure 1:**
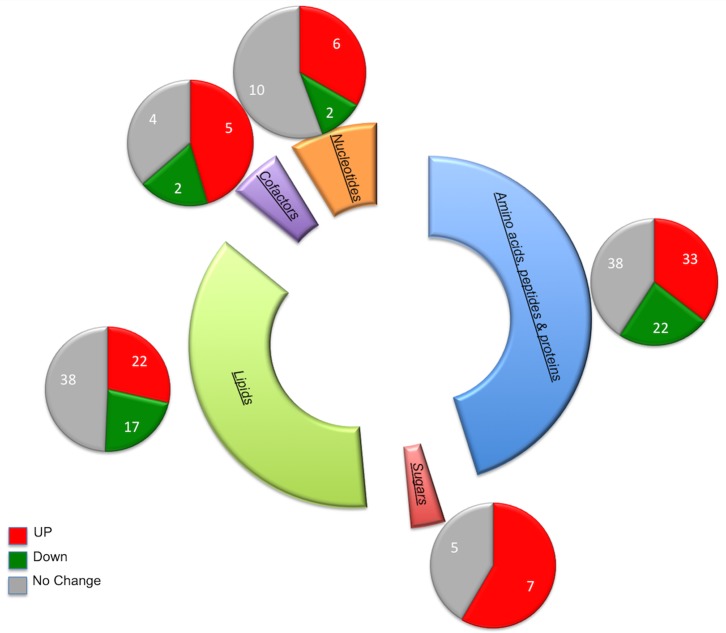
Distribution of the metabolomics data with colors depicting the levels of metabolites.

Probing the metabolome of 50 paired skin samples obtained from sun-exposed and sun-protected sites, through mass spectrometry, we quantified a total of 241 metabolites. We performed imputation with minimum observed values for each compound with the missing values. A paired t-test was performed to identify metabolites that differed significantly between the biopsies extracted from sun-exposed and sun-protected skin respectively. A subset of 122 metabolites was significantly different (p-value <0.05) with a false discovery rate threshold of less than or equal to 5% between the two sets of samples. Out of the 122 metabolites, 46 were lower and 76 were higher in biopsies extracted from sun-exposed sites compared to biopsies from sun-protected regions. The identified metabolites belonged to a total of 52 biological pathways and a subset of 42 pathways had one or more metabolite(s) significantly different when biopsies obtained from sun-exposed regions were compared to the one obtained from sun-protected regions. These pathways spanned amino acids, nucleotides, sugars, peptides, cofactors, lipid metabolism and others (Figure 1).

**Figure 2:**
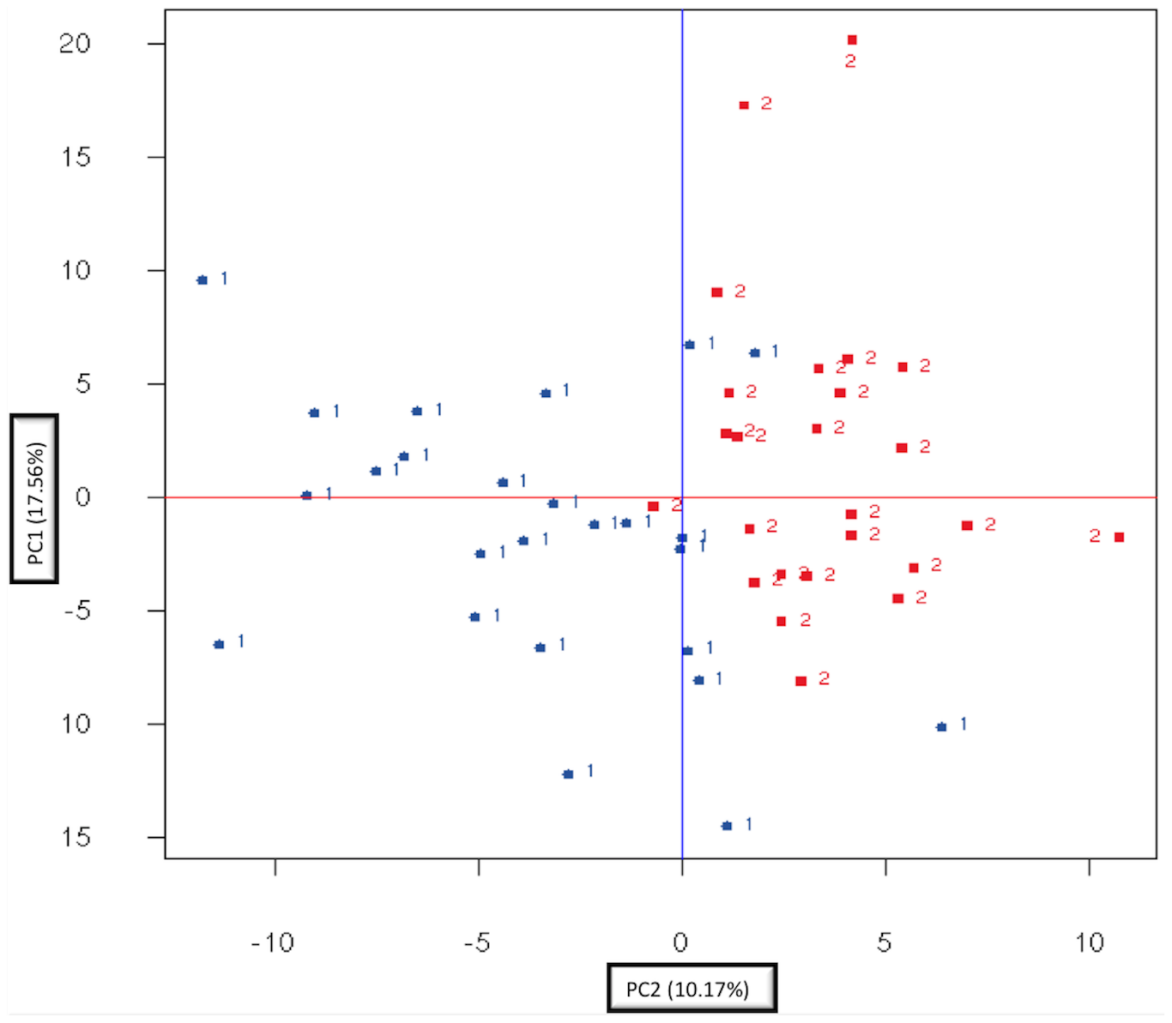
Principal component analysis (PCA). PCA plot showing separation of the sun-exposed and sun-protected skin samples. 1- unexposed inner arm. 2- exposed outer arm.

Comparing the global metabolite pattern in the biopsies obtained both from sun-exposed and sun-protected sites through principal component analysis; demonstrated clearly that sun exposure altered the metabolic profile in the sun-exposed skin biopsies (Figure 2).

**Figure 3:**
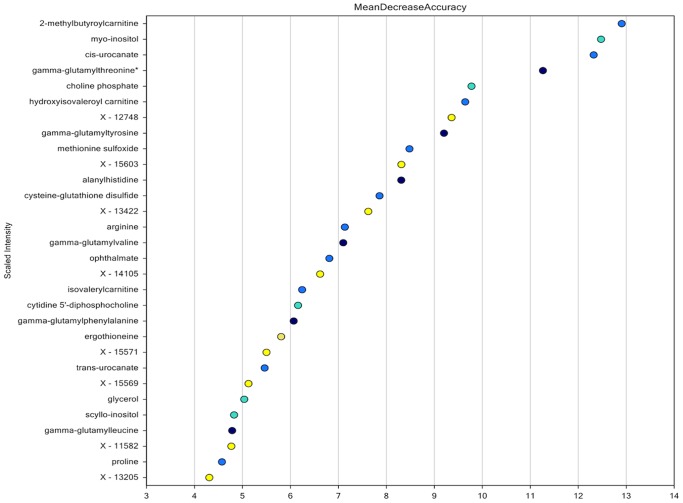
Random forest classification. Random forest classification presenting the prioritized list of 30 metabolites. Metabolites with X- prefix are unknown and need to be identified. Y-axis is the prioritized list in which we as move downward, there is decrease in the accuracy (X-axis) of classification of the two samples.

In this study, significant alterations in multiple classes of metabolites were observed pointing to comprehensive alteration in the metabolic profile of sun-exposed skin (Table 1). In order to develop a metabolomic signature that can be used to determine the photo exposure status of skin. A Random Forest classification was performed on the entire data set (including significant and non-significant metabolites). This analysis produced a list of 30 metabolites ranked in order of their importance to classify accurately the exposed and unexposed skin samples (Table 2). The prioritized list of metabolites is listed in Figure 3, denoted as the biochemical importance plot. This prioritized list is the metabolomics signature, which can classify the sun-exposed and sun-protected skin samples with very high accuracy. As expected, in this signature, cis-urocanate; a validated biomarker for UV damage [16], [17] had the third most reduction in classification error. Additionally, the prioritized metabolite list presented a theme of metabolomic catabolism and oxidative stress as a result of sun exposure between the two classes, which was further corroborated when we analyzed the three major networks. Most of these pathways suggest increased production of reactive oxygen species (ROS), which resulted in increased oxidative stress that can be held responsible for changes in the phenotypic appearance of sun-exposed skin.

**Table 1:** Summary of the Biochemical Pathways and Compounds altered in sun-exposed sites.

Pathway	Compounds	Ratio	p-value
**Purine Metabolism pathway**	Inosine	Increased	0.03
	Xanthine	Increased	0.04
	Hypo-xanthine	Increased	0.04
	Uric acid	Increased	0.01
	Adenosine	Decreased	0.02
	Guanosine	No change	
**Methionine and glutathione Metabolism pathway**	S-adenosylhomocysteine	Increased	<0.001
	Cysteine	Increased	<0.001
	Reduced glutathione	Decreased	0.002
	Cysteine-glutathione disulphide	Increased	<0.001
	Glycine	No change	
	Glutamate	No change	
	Gamma-methyl ester	Decreased	0.01
	gamma-glutamylvaline	Decreased	<0.001
	gamma-glutamylleucine	Decreased	<0.001
	gamma-glutamylisoleucine	Decreased	0.03
	gamma-glutamylglutamate	Decreased	0.001
	gamma-glutamylphenylalanine	Decreased	<0.001
	gamma-glutamyltyrosine	Decreased	<0.001
**Nicotinamide Metabolism pathway**	Nicotinamide	Increased	0.05
	Nicotinamide ribonucleotide	Increased	0.02
	Nicotine riboside	Increased	0.05
	Quinolinic acid	Not found	
	Nicotinic acid	Not found	
	Mononucleotide	Not found	
	Nicotinamide adenine dinucleotide	No change	

Increased: The metabolites were up-regulated in biopsies from sun-exposed skin.

Decreased: The metabolites were down-regulated in biopsies from sun-exposed skin.

**Table 2:** The classification accuracy.

Random Forest		Predicted Group		Class Error
		Sun-protected	Sun-exposed	
**Actual Group**	Sun-protected	24	1	0.04
	Sun-Exposed	0	25	0

The table represents the classification accuracy with perfect classification in Sun-exposed and very high accuracy in Sun-protected skin samples using the prioritized list of 30 metabolites through Random Forest Classification.

#### Adenosine catabolism leading to production of higher uric acid

**Figure 4:**
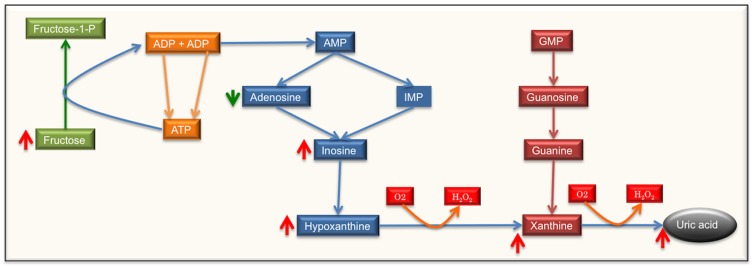
Adenine metabolism. This schematic presents the steps in the adenosine and guanosine pathway leading to production of uric acid. ATP – Adenosine triphosphate, GMP – guanosine monophosphate, IMP – inosine monophosphate. Red arrows – higher accumulation, green arrow – low accumulation.

We observed significantly higher levels of inosine, inosine monophosphate, xanthine, hypo-xanthine, uric acid without any change in adenosine level. These metabolites belong to the Adenosine degradation pathway in which adenosine is deaminated to form inosine that is converted into hypo-xanthine, xanthine and is further converted into uric acid. Another purine, guanosine's degradation can also produce xanthine however; there was no significant difference in the levels of intermediate guanosine degradation metabolite; guanine, when the two sets of metabolomes were compared (Figure 4). These data suggest that purine degradation is limited to adenosine and there is no contribution to purine catabolic products through guanine degradation branch of the pathway. The contribution of guanosine catabolic products to production of uric acid needs further investigation.

The Purine degradation pathway has been suggested as a one of the major biochemical source for ROS production [18] and in this case can be hypothesized as one of the main pathways fueling high oxidative stress in sun-exposed skin. Xanthine oxidase, the main enzyme in this pathway, degrades xanthine to hypoxanthine and further to hydrogen peroxide, and has been previously reported to produce ROS [19]. Previous findings have shown increased activity of this enzyme in epidermis after exposure to UVB [20], [21]. In particular UV-mediated oxidative stress activates xanthine oxidase, resulting in generation of superoxide ions. Additionally, activity of xanthine oxidase has been considered as a major factor in UVB-induced antioxidant consumption and depletion [21] and inhibition of this particular enzyme by inhibitors like oxypurinol has been shown to decrease ROS generation. In this study increased ratios of hypoxanthine and uric acid suggest increased activity of xanthine oxidase, which can be hypothesized as one of major factors contributing toward the phenotypic appearance of sun-exposed skin.

Interestingly, we also measured significantly higher levels of fructose in the exposed skin samples. Brosh *et al*. [22] had reported that higher consumption of fructose could lead to higher degradation of adenosine in the liver. However the source of simple sugar such as fructose in this study is not clear. Accumulation of fructose could be the result of carbohydrate degradation through UV exposure or could be contributed by diet as well. The exact mechanism through which higher level of fructose plays a role in adenosine catabolism in catabolic pathway needs further investigation.

Taken together, our results suggest that UV exposure is leading to degradation of adenosine and potentially contributing towards a more oxidized state in the skin. It will be interesting to investigate the cause and effect relationship between high sugar diet and adenosine catabolism and subsequently in photo aging.

#### Altered homocysteine pathway leading to altered ratio of glutathione

**Figure 5:**
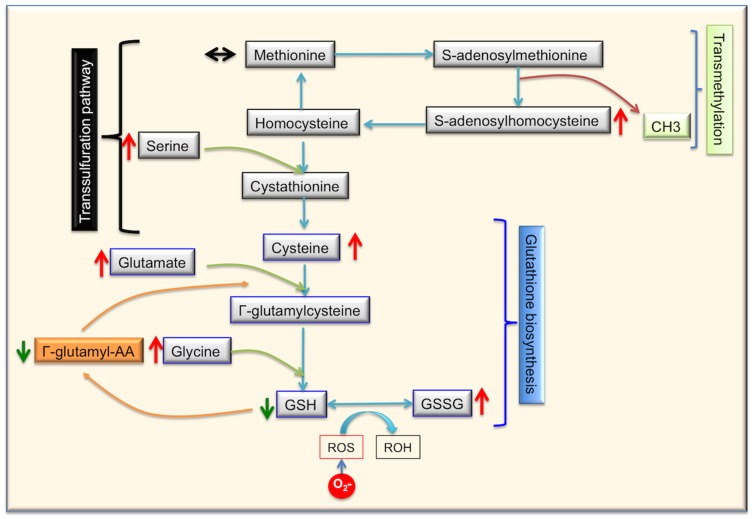
Methionine-Glutathione metabolism. This figures details the levels of various metabolites that were detected in the pathaway. Red arrow indicates higher levels, green arrow indicates lower levels and black arrow indicates no change in the levels of the metabolite.

The Methionine and glutathione pathways are connected by the transsulfuration pathway in which methionine cycle provides sulfur for cystathione formation through homocysteine [23] (Figure 5). In the methionine pathway, we measured no significant change in the levels of methionine, S-adenosylmethionine and homocysteine. Only S-adenosylhomocysteine had a significantly higher accumulation in the sun-exposed skin samples as compared to sun-protected skin samples. However, metabolites further than the transsulfuration pathway such as cysteine, GSH, GSSG were measured significantly different between the sun-exposed and sun-protected skin samples. The ratio of glutathione (GSH) to oxidized glutathione (GSSG) was lower in the sun-protected samples, suggesting increased oxidative stress. High levels of Cysteine-glutathione disulfide and the low ratio of GSH to GSSG reflect prevalence of oxidizing conditions in sun-exposed skin samples. Additionally, high levels of cysteine, glycine (not statistically significant) and glutamate, gamma-methyl ester were also detected. These metabolites are part of the glutathione biosynthesis pathway. Both of these scenarios would result in decreased glutathione levels in skin and would reflect the pervasiveness of oxidative stress in photo exposed skin.

A biological system can also utilize an alternate pathway to generate glutathione. In this pathway, glutathione biosynthesis is achieved through γ-glutamylaminoacids, 5-oxoproline and glutamate. We found that the levels of various γ-glutamylaminoacids (γ-glutamylalanine, γ-glutamylleucine, γ-glutamylisoleucine, γ-glutamylphenylalanine and others) were detected at significantly low levels in the sun-exposed skin samples as compared to the sun-protected skin samples. These findings indicate that the alternate pathway involving γ-glutamylaminoacids is working at a lower level as compared to the pathway involving cysteine and glycine.

Collectively, GSH/GSSG definitely points towards increased level of ROS and oxidative stress and has been documented in photo exposed skin [24]. According to the free radical theory of aging, ROS increases with aging due to the reduced activity of the antioxidant defense enzymes [25]–[27], similarly in this case it could be hypothesized that enzymatic machinery might be modulated resulting in high oxidative stress. Further investigation of the activity of enzymes such as transpeptidase or dipeptidase involved in the methionine-glutathione pathways, after UV exposure and oxidative stress may clarify the role of these enzymes in photoaging.

#### Nicotinamide pathway suggests skin is using salvage pathway as compared to de novo production to consume the damaged nicotinamides

**Figure 6:**
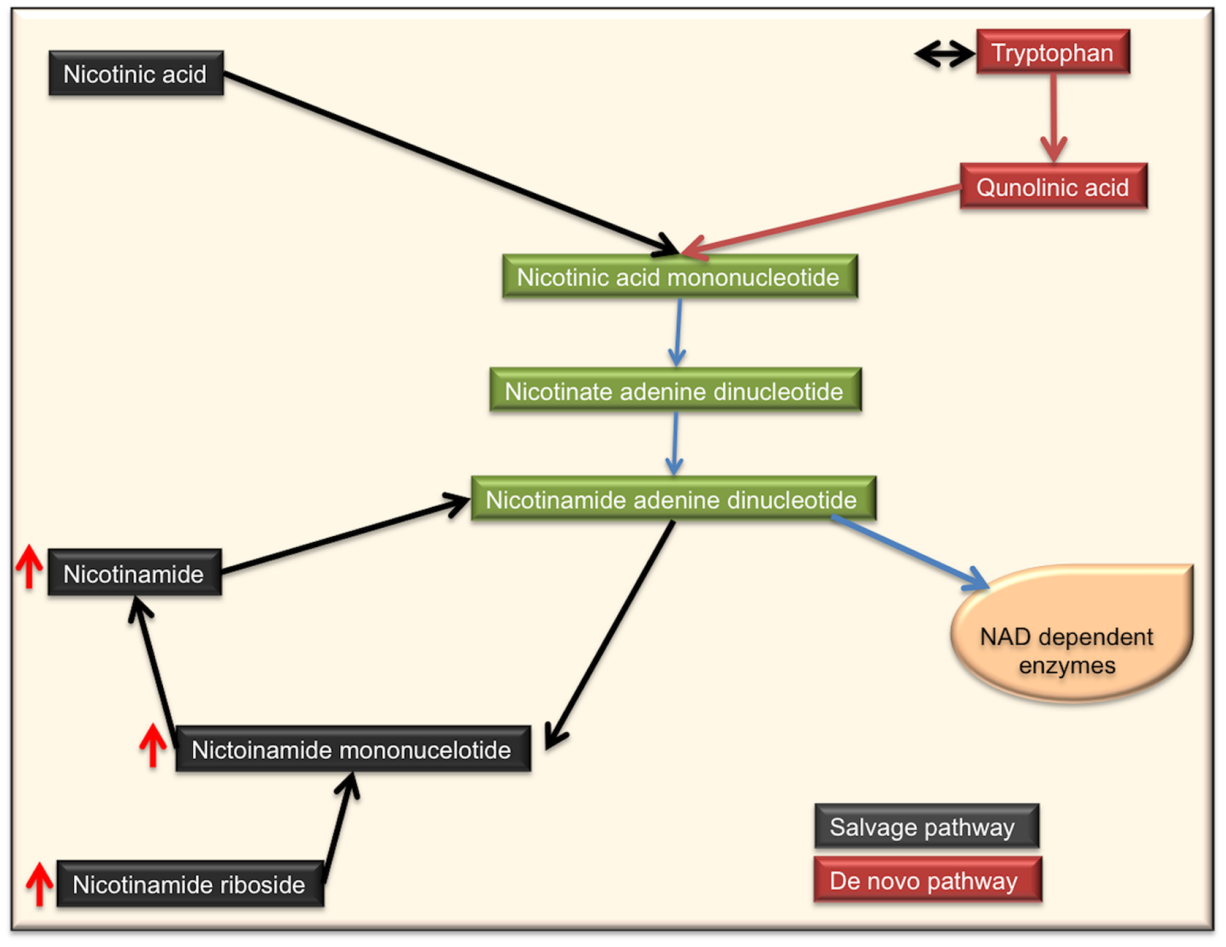
Nicotine metabolism pathway. This schematic presents the de novo and salvage pathways through which the NAD can be generated. Red arrow indicates higher accumulation and black arrow indicates no change.

While studying sun-exposed skin we identified three NAD+ metabolites; nicotinamide adenine dinucleotide (NAD), nicotinamide ribonucleotide (NMN) and nicotine riboside (NR), which were significantly increased compared to sun-protected skin (Figure 6). NR had highest fold change (2.69) in the exposed samples as compared to the unexposed. All of these metabolites belong to the salvage pathway and their higher accumulation indicated hyperactivity of NAD salvage pathway in sun-exposed skin samples. The biosynthesis of NAD+ occurs through salvage and/or *de novo* pathways. In the *de novo* pathway, NAD is synthesized from tryptophan and in the salvage pathway NAD+ is synthesized by reclaiming degradation products of metabolites having nicotinamide ring NAD [28]. We did not detect either quinolinic acid or nicotinic acid mononucleotides which are critical intermediates in the de novo synthesis of NAD and their absence might indicate hypoactivity or no activity of NAD production through *de novo* synthesis.

Nicotinamide pathway has been studied extensively and its respective metabolites have been shown to influence cellular processes including DNA repair, genomic stability, the immune system, stress responses, signaling, transcription, apoptosis, metabolism, differentiation, chromatin structure and life span [29]. In addition to its well-known redox functions in energy metabolism, NAD and NADP are also required for the synthesis of cyclic ADP-ribose and NADP, which are two major mediators of intracellular calcium signaling pathways [29]. These processes indicate a theme of degradation in the cell because the salvage pathway recycles the degraded products of nicotinamide containing metabolites. Despite higher biosynthesis of NAD+, we did not see a significant change in the NAD+ levels between the two sets of samples. These data suggest that all the NAD+ produced through salvage pathway is siphoned to produce raw materials for DNA, proteins and other cellular processes. Furthermore the UV induced cutaneous proliferation does justify the need for increased amount of genetic material for new multiplying cells [30]; however the increased demand of energy by the biological system cannot be ignored at the same time, hence salvage pathway being more energy efficient and meets the current needs of the biological system. Additionally, these findings completely agrees with previously published studies that reported an increased glycolytic pathway for energy production instead of TCA cycle in sun-exposed skin [24].

## Conclusions

Chronic exposure of skin to sun results in phenotypic changes referred as photoaging. Photoaging has been, associated with inflammation, which could be due to or result in high oxidative stress in skin that accelerates the mechanism of aging. The effect of sun exposure on skin has been widely studied both from genomic and proteomic point of view and very well correlated to phenotypic appearance of sun-exposed skin; however, metabolites, the end products produced as a result of these pathways are discussed as a part or end result of a particular condition or pathways. These particular metabolites discussed in scientific literature can provide limited information about that particular pathway or condition, whereas the whole process of transformation in sun-exposed skin from metabolite point of view cannot be judged. Here we present the first study which profiled a wide range of metabolites and identified a highly accurate metabolomic signature of sun exposed skin. We also detailed the effect of sun exposure on the biological processes using the metabolomics data generated through mass spectrometry.

From a given set of metabolites, we found that the skin is under high oxidative stress and the chains of reactions to produce these metabolites are inclined toward catabolism rather than anabolism. These catabolic activities presuade the skin cells to generate metabolites through the salvage pathway instead of *de novo* synthesis pathways. Additionally, UV induced metabolic catabolism produces higher oxidative stress which in turn leads to recursive catabolism and higher oxidative stress. The observations suggest that skin tissue is trying to cope up with the stressful conditions by using simpler and less energy involved reactions. This is in agreement with our earlier published findings [24]. The findings raise new questions regarding energy metabolism in photo-aged skin and definitely warrants further research in this particular area of interest.
